# Evaluation of CD133 and CD56/NCAM expression in Wilms tumor and their association with prognostic factors

**DOI:** 10.22038/ijbms.2020.41468.9804

**Published:** 2020-07

**Authors:** Amir Hossein Jafarian, Nona Zabolinejad, Nema Mohamadian Roshan, Sara Hashemi, Masoumeh Gharib

**Affiliations:** 1 Department of Pathology, Faculty of Medicine, Mashhad University of Medical Sciences, Mashhad, Iran

**Keywords:** CD133, Immunohistochemistry, Neural cell adhesion – molecules, Stem cells, Wilms tumor

## Abstract

**Objective(s):**

To validate certain markers for cancer stem cell populations and their clinical importance in Wilms tumor (WT)

**Materials and Methods:**

Immunohistochemical study for CD133 and CD56/NCAM was performed on forty-six cases of WT that were diagnosed between 1999 and 2015, and the association of these markers with survival and prognostic factors was analyzed.

**Results:**

Thirty-four (73.9%) of WTs were positive for CD133 and thirty-nine (84.8%) were positive for CD56/NCAM. A significant positive correlation between CD133 and CD56/NCAM expression and the National Wilms Tumor Stage (NWTS) and death was found. Moreover, overall survival time was significantly correlated with CD133 and CD56/NCAM H-score, NWTS stage, and death.

**Conclusion:**

It seems that CD133 and CD56/NCAM expressions can be used as strong prognostic parameters for the survival of patients with WT and can be used to predict WT patients’ stage. Moreover, their targeted therapies can abolish cancer stem cells in children with recurrent tumors.

## Introduction

One-hundred fifty years ago, cancer stem cell theory was hypothesized to reveal the existence of a fraction of tumor cells with stem cell-like properties (1). These stem cell-like tumor cells are insensitive to conventional chemo/radiotherapy and this poses a problem in therapy. Some of these cells can be resistant to antitumor drugs and therefore lead to the formation of secondary tumor foci with stronger drug resistance. Hence, research on cancer stem cell (CSC) targeted therapies and production of drugs with the ability to reverse CSC drug resistance is blooming (2). 

The incidence of Wilms tumor (WT) in North American white children is 1/10,000 (3,4). Over the last 40 years, there has been a rise in WT survival rates (from <30% to 85–90%), which is attributed to innovations in therapy (5); nonetheless, the adverse effects of chemo/radiotherapy is tremendous and leads to secondary malignant tumors, morbidity, and mortality. One of the options to evade these outcomes would be to develop targeted therapies against these CSCs. Yin *et al*. first found CD133 (prominin-1) protein, which has a 120 kDa molecular weight, on hematopoietic stem cells. It has a membranous localization (6). So far, researchers have found CD133 as a CSCs marker in numerous neoplasms (7-10).

CD56/NCAM is found to be expressed on some of the normal cells and is found in some neoplasms (11, 12). Its targeted therapy is a drug conjugate by the name of IMGN901 (lorvotuzumab mertansine), which can be beneficial in CD56/NCAM positive tumor therapies (13, 14).

According to the aforementioned information and regarding the fact that WT is one of the major renal cancers of pediatric age, we decided to perform a retrospective cross-sectional study with the main purpose of investigating the expression of CD133 and CD56/NCAM in WT and their association with prognostic factors and survival.

## Materials and Methods

**Figure 1:**
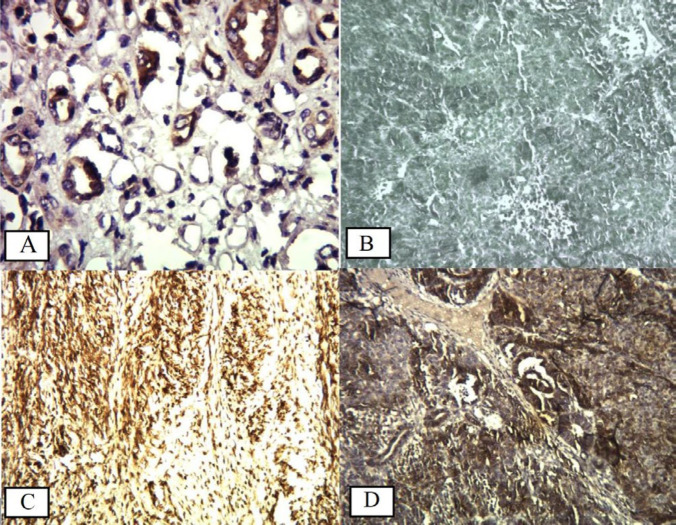
CD133 staining in Wilms tumor. A: kidney tubules, B: Negative staining (100×), C: Moderate staining in the mesenchymal cells (50% of the cells) (100×), D: Strong staining in 80% of blastemal and epithelial cells (100×)

**Figure 2:**
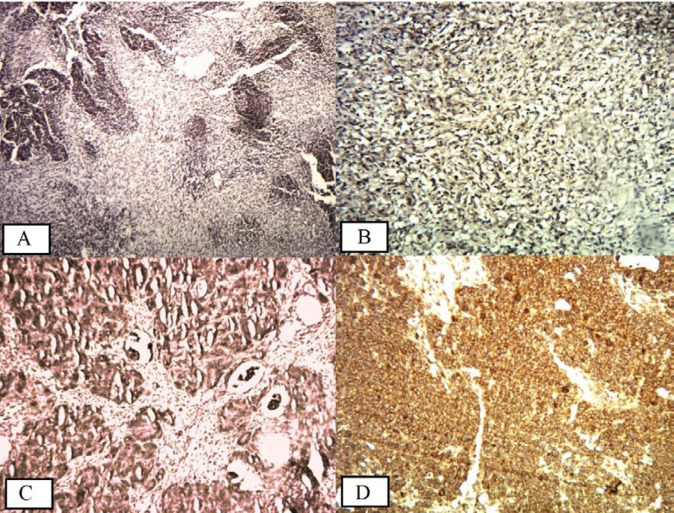
A: Negative staining of CD56/NCAM in the blastemal and mesenchymal cells of Wilms tumor (40×), B: Mild staining in 80% of mesenchymal cells in Wilms tumor (100×), C: Moderate staining in 75% of the epithelial and blastemal cells in Wilms tumor (100×), D: Strong staining in 80% of blastemal cells in Wilms tumor (400×)

This was a retrospective cohort study on formalin-fixed, paraffin-embedded (FFPE) tissue samples of patients with WT who were diagnosed and treated between 1999 and 2015 at a major tertiary referral center. We included all possible available patients so there was no certain sampling strategy in this research. Hematoxylin and eosin (H&E)-stained slides, pathology and other medical records were assessed to confirm the diagnosis, and clinicopathologic parameters, such as demographic data, size of the neoplasm, differentiation, anaplasia, and tumor stage were gathered. Based on the largest series studied in Iran by Mehrazma (15), it was expected to have an approximate intensity of 87% for CD133 positivity in WT which yielded a total sample size of forty-five according to the following formula: Sample size = Z2*p*(1-p) / C2, where Z is the critical value of the Normal distribution, C is the margin of error, and p is the sample proportion. Positive and negative controls were used to reduce the effects of any confounders. Glioblastoma was the positive control for CD133, and small cell lung carcinoma was the positive control for CD56/NCAM. Negative control was assessed by not adding the primary antibody. Immunohistochemical staining was performed on 5-μm tissue sections using primary antibodies of rabbit polyclonal anti-CD133 (Biorbyt, UK) and mouse monoclonal anti-CD56 (clone 1B6, Novocastra, Newcastle, UK). All of the immunostained slides were scored semi-quantitatively by two expert pathologists without any knowledge of the clinicopathologic parameters and the outcome of the patients. The pathologists blindly assessed the specimen and were not aware of the outcomes. There were two pathologists and any discrepancy was assessed in a joint meeting to reach a consensus. The slides were scanned to investigate tumor cells’ overall distribution (Nikon light microscope, Japan, 10×) and semi-quantitative final scores were given at higher power. The staining outcomes were assessed as the intensity on a scale of 0 to 3 as absent, weak, moderate, or strong, and semi-quantitative assessment of positive tumor cell percentage, which was on a scale of 0 to 4 as 0 (0 -10%), 1 (11-25%), 2 (26-50%), 3 (51-75%), or 4(76-100%). Histochemical score (H-score) of staining was calculated by adding these two variables and a final score of 0 to 7 was rendered. The cut-off value was specified by Receiver Operating Characteristic (ROC) curve (SPSS16) to classify the samples as negative or positive. Accordingly, H-score 0, 1, 2 and H-score 0, 1, 2, 3, 4 were counted as negative for CD 133 and CD56/NCAM and H-score 3, 4, 5, 6, 7 and H-score 5, 6, 7 were counted as positive for CD 133 and CD56/NCAM, respectively. It should be mentioned that CD133 staining was also seen in the normal kidney tubular epithelium. Glomeruli and interstitium were CD133 negative. The normal kidney was CD56/NCAM negative (Figures 1 and 2). Moreover, forty-six WT patients were followed up. The survival time was assessed from surgery to the last follow-up or recurrence, metastasis or death. 

***Statistical methods***

Statistical analysis consisted of both descriptive statistics and analytical tests. Descriptive statistics of the data included mean and percentage and for analytical tests, Pearson’s chi-square and Fisher’s exact tests were used for univariate analysis to determine the associations between the prevalence of CD133+ and NCAM+ tumor cells and each clinicopathologic parameter. Patient survival time was determined using the Kaplan and Meier method with log-rank analysis. There was no missing data as the patients were completely cooperative in giving pathological and other required tests, neither was missing data in their clinical records.

## Results

Forty-six FFPE tissue specimens with a definite diagnosis of WT were analyzed. This project yielded the following results: the clinicopathological characteristics of 46 patients with WT are shown in Table 1. Age ranged from 3 to 108 months (mean=39 months) and male to female ratio was 1:1. The range of the tumor size (in greatest diameter) was from 4 to 26 cm with a mean of 10.4 cm. The range of the tumor weight was from 23 to 2300 with a mean of 567 g. Thirty-five (76.1%) patients were at low stage, while eleven (23.9%) were at high stage. There was no report of any relapse. Thirty-nine patients were alive after a median follow-up of 79 months and five were dead.

CD133 intensity showed positive staining in 73.9% (34/46) of specimens, with mild staining in twenty-five (54.3%) and moderate staining in nine (19.6%) samples. Seven (15.2%) tumors showed extensive expression (more than half), whereas thirty-nine (84.8%) samples demonstrated immunoexpression in less than half of cells. H-score estimate showed twenty-eight out of 46 (60.9%) as CD133 negative (H-score ≤3), while 39.1% of cases (18/46) were considered positive (H-score >3).

In terms of CD56/NCAM intensity, 84.8% (39/46) of the tumors revealed membranous staining with ten (21.7%) tumors as weak, twenty-one (45.7%) as moderate and eight (17.4%) as strong intensity. CD56/NCAM-staining showed thirteen (28.3 %) tumors with extensive expressions (more than half of cells), whereas thirty-three (71.7%) samples demonstrated CD56/NCAM staining in less than half of tumor cells. By using the H-score method and five as the Cut-off value, 33 (71.7%) were CD56/NCAM negative (H-score <5), and 13 (28.3%) were CD56/NCAM positive (H-score>5). The correlation between CD133 and CD56/NCAM expressions and clinicopathological factors in WT is summarized in Table 2.

**Figure 3:**
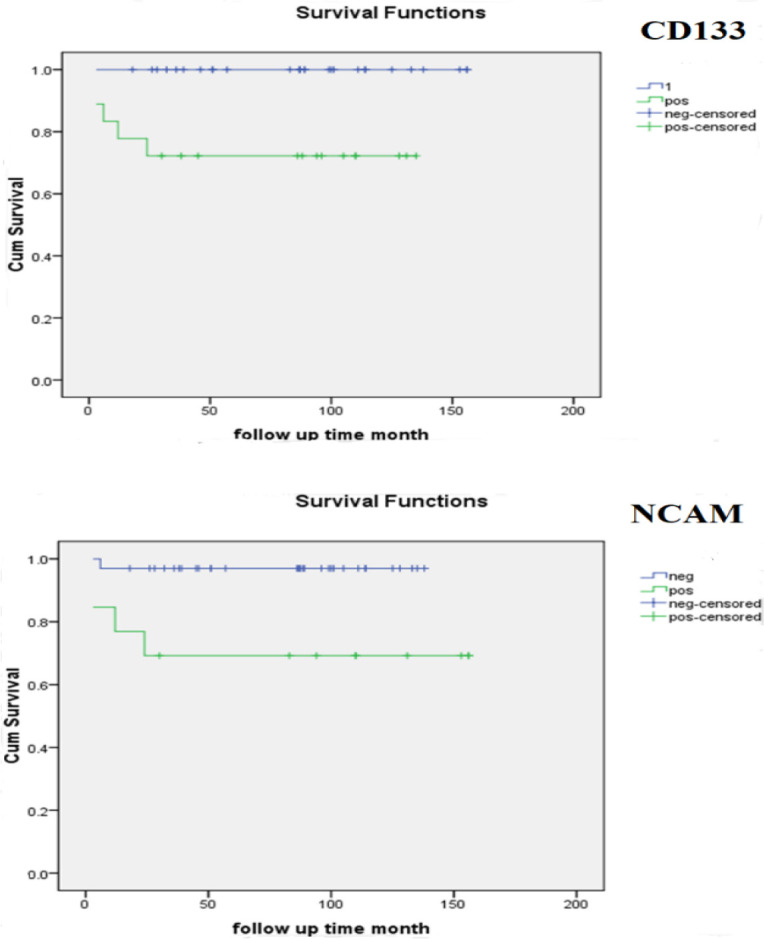
Correlation between CD133 H-score and CD56/NCAM H-score and overall survival time of Wilms tumor

Kaplan-Meier estimation of overall survival for all WT patients was 140.01±6.75 months. The overall survival rate of CD133 and CD56/NCAM positive and CD133 and CD56/NCAM negative patients was significantly different (Figure 3). The overall survival rate for low-stage tumors is 147.82±5.61 months and for high-stage tumors is 99.81±17.33 months, which is statistically significant (Log-rank Mantel-Cox: *P*-value=0.037). There was no statistical association between the overall survival time and gender, age, and histological type.

## Discussion

Thanks to improvements in modes of therapy, some adult tumors are dealt with targeted therapy against CSC. This raises the question of whether or not CSC exists in pediatric tumors and whether they act similarly. A positive answer to this question would lead to a new path of targeted treatment in this age group. Moreover, research data emphasize the intimate relation of CSC and relapse and metastasis of some neoplasms and their drug resistance. Therefore, CSC can be a prognostic factor.

The first challenge encountered is how to differentiate CSC from tumor cells. One solution is to find cell markers that are only expressed in these cells and hereby demolishing the tumor by targeted therapy. So far, CSCs were distinguished by markers such as CD133 and CD56/NCAM. The other challenge is how to detect and quantify CSC in solid tumors, which are less accessible, compared to leukemia.

Little data about the expression of these CSCs in WT and its clinical and prognostic significance prompted us to conduct this study. We studied the expressions of two CSC markers (CD133 and CD56/NCAM) in WT by immunohistochemistry and then their relationship with prognostic factors and survival was analyzed.

There were 23 (50%) male and 23 (50%) female patients, and the ratio of male to female patients was 1:1. This is in accordance with the literature (3, 4, 16). Patients’ ages ranged from 3 to 108 months (mean = 39 months). Based on the NWTS classification (17), out of forty-six WT patients, there were twelve (26.1%) at stage I, twenty-three (50%) at stage II, seven 15.2%) at stage III, three (6.5%) at stage IV, and only one (2.2%) at stage V. 

There are multiple studies on the association of CD133 expression with poor prognosis in colorectal cancer (18), pancreatic cancer, hepatocellular carcinoma (19), neuroblastoma (20), and in patients with glioma (21). Zeppernick, *et al*. showed that the presence of CD133+/Ki67+ cells in glioblastomas is an independent predictor for patients ‘survival (21). Other studies did not consider it as prognostic markers in the pancreas and liver (19). Choi, *et al*. studied on colorectal cancer and showed CD133 relation to invasiveness and differentiation of the tumor but not with patients’ survival (22). On the other hand, there are studies suggesting it to be a favorable prognostic marker in some tumors like clear-cell renal cell carcinoma in which a high degree of CD133 expression is related to more differentiated morphology and non-metastatic disease (8). Therefore, it implies that it does not predict poor prognosis for every neoplasm. To our knowledge, there are few studies on the clinical significance of CD133 expression in WT. In 2012, Mehrazma, *et al.* found a positive correlation between CD133 staining and NWTS (15). 

We found that WTs were positive for CD133 in 39.1% of the cases. H-score revealed a positive correlation (*P-value*=0.014) between the CD133 expression and NWTS (The CD133 expression was observed in 55.6% of WTs at lower stages, whereas only 44.4% of higher stage tumors were positive for CD133). This is in agreement with Mehrazma’s study(15). A higher expression of CD133 (H-score) was also more often seen in dead patients with WT compared to live patients (*P-value*=0.006). However, there was no correlation between CD113 expression and sex, age, anaplasia, epithelial differentiation, or histology. One potential explanation for these observations could be attributed to the limitation on sample size.

Ash *et al*. used CD56/NCAM as a means to distinguish Ewing-sarcoma patients with excellent prognosis from those with poor prognosis, hence implementing personalized therapy (23). Atsunori Tsuchiya, *et al*. considered its soluble status as a significant independent prognostic marker in hepatic cell carcinoma patients, and stated that high soluble CD56/NCAMlevels were significantly related to intrahepatic metastasis (24). In 2013, Naomi Pode-Shakked, *et al*. established for the first time a WT xenograft model simulating an aggressive tumor and showed that by targeting a certain population of cancer cells (enriched in CSC activity i.e.CD56/NCAM), dramatic eradication or attenuation of the tumor can occur in xenograft models (2). These invaluable studies show the potential prognostic significance of this marker. We found that WTs were positive for CD56/NCAM in 28.3% of cases. In the univariate analysis, the expression of CD56/NCAM was positively correlated with the NWTS stage of WTs (*P-value* =0.006), indicating that high stage tumors were more likely to express CD56/NCAM when compared to low stage ones. As in CD133, higher expression of CD56/NCAM was seen in dead patients rather than in live ones (*P-value*=0.01). These findings are in concordance with those of other studies. However, there was no significant correlation between the expression of CD133 as assessed by H-score and sex, tumor histology, tumor anaplasia, or patient’s age, which could be attributed to low sample size. Moreover, these forty-six patients with WT were followed up and it was shown that there is no relationship between the median survival time and histological type, which could be related to sample size. However, the median survival time was significantly correlated with CD133 and CD56/NCAM H-score, NWTS stage, and death. The median survival time of CD133 and CD56/NCAM negative patients was longer than that of CD133 and CD56/NCAM positive patients. Moreover, the five year survival time was 84.84%, which is similar to the five-year survival in the literature (80-90%) (17).

Similar to other studies, we found that in WT, the amount of cancer cells expressing CD133 and CD56/NCAM is far too high to be limited to a cancer stem cell population. One of the limitations was that a cancer cell may express one of these markers but does not function as a CSC and vice versa. The other limitation of this study was those specimens that did not fulfill inclusion criteria and were not included in the study and more proper archiving strategies and better follow-up could be very helpful. Wilms tumor is a very rare tumor of childhood and even though we studied all the cases of this tumor retrospectively in 10 years, further studies on bigger sample size are recommended. 

**Table 1:** Clinicopathological characteristics of 46 patients with Wilms tumor

Variable	No. of patients (%)	Variable	No. of patients (%)
Age (months)		**Epithelial pattern**	
≤24 >24	20 (43.5%) 26 (56.5%)	**Homologous** **Heterologous**	45 (97.8%) 44 (97.8%) 1 (2.2%)
Gender		**Mesenchymal pattern**	
Male Female	23 (50%) 23 (50%)	**Homologous** **Heterologous**	45 (97.8%) 36 (80%) 9(20%)
Associated anomalies			
Undescended testicle Bifid rib/ mental retardation Horseshoe kidney	3 (6.5%) 1 1 1	**Nephrogenic rest** **Absent** **Single** **Multiple**	43(93.5%) 1(2.2%) 2(4.3%)
NWTS stage		**Invasion**	
Stage I Stage II Stage III Stage IV Stage V	12(26.1%) 23(50%) 7(15.2%) 3 (6.5%) 1 (2.2%)	**Absent** **Capsular** **Sinusal soft tissue** **Sinusal soft tissue and vessel** **Capsular and sinusal** **Ureter**	14 (30.4%) 10 (21.7%) 10 (21.7%) 3 (6.5%) 8 (17.4%) 1 (2.2%)
Histology			
Triphasic Biphasic	44 (95.7%) 2 (4.3%)	**Lymph node involvement** **Free** **Metastatic**	9 (84.8%) 7(15.2%)
Blastemal pattern			
Organoid Diffuse Both	46 (100%) 29 (63%) 8(17.4) 9(19.6)	**Histology** **Favorable** **Unfavorable**	43(93.5%) 3(6.5%)
Laterality			
Right Left Bilateral	20 (43.5%) 25 (54.3%) 1(2.2%)		

Moreover, as thirty-three patients had WT for sixty months (five years), and as there were five deaths in this group, the 5 year survival time could be calculated, which was 84.84%

**Table 2:** Association between CD133 and CD56/NCAM expression and clinicopathologic factors in 46 patients with Wilms tumor

	n	Expression of CD133		*P-value*	Expression of CD56/NCAM		*P-value*
		Negative	Positive		Negative	Positive	
Age (months)							
≤24 >24	20 26	14(50) 14(50)	6(33.3) 12(66.7)	0.36	16(48.5) 17(51.5)	4(30.8) 9(69.2)	0.33
Gender							
Male Female	23 23	16(51.7) 12(42.9)	7(38.9) 11(61.1)	0.22	16(48.5) 17(51.5)	7(53.8) 6(46.2)	0.74
Histology							
Favorable histology Unfavorable histology	43 3	27(96.4) 1(3.6)	16(88.9) 2(11.1)	0.55	31(93.9) 2(6.1)	12(92.3) 1(7.7)	1.000
Epithelial pattern							
Homologous Heterologous	44 1	26(96.3) 1(3.7)	18(100) 0(0)	1.000	31(96.9) 1(3.1)	13(100) 0(0.0)	1.000
NWTS stage							
Low- stage High-stage	35 11	25(89.3) 3(10.7)	10(55.6) 8(44.4)	0.014	29(87.9) 4(12.1)	6(46.2) 7(53.8)	0.006
Status							
Dead Alive	5 41	0(0) 28(100)	5(27.8) 13(72.2)	0.006	1(3.0) 32(97.0)	4(30.8) 9(69.2)	0.018

## Conclusion

Our data clearly shows that CD133 and CD56/NCAM expression in WT may be useful to predict WT patients’ stage and prognosis, which can be useful for precision medicine. Moreover, their expression seems to be a strong prognostic parameter for the survival of patients with WT. Therefore, CD133 and CD56/NCAM expression analysis can identify prognostic groups. Further usage of multiple CSC immunomarkers on larger sample size and assessment of these parameters in an in vivo setting could be more informative.
